# Household food insecurity, low maternal social support and maternal common mental disorders in East Mamprusi Municipality, Ghana

**DOI:** 10.1186/s12889-023-16157-x

**Published:** 2023-06-28

**Authors:** Anthony Wemakor, Mohammed Bukari, Raymond Atariba

**Affiliations:** Department of Nutritional Sciences, School of Allied Health Sciences, University for Development Studies, P. O. Box TL 1883, Tamale, Ghana

**Keywords:** Household food insecurity, Social support, Common mental disorders, Mothers, East Mamprusi, Ghana

## Abstract

**Introduction:**

Both household food insecurity and maternal common mental disorders are public health concerns in Ghana but studies on them, and their interrelatedness are scarce. Social support is an independent determinant of mental health but can also moderate the link between risk factors and mental illness. Identifying the risk factors of mental illness may provide opportunities for intervention and help reduce disease burden and impact. This study examined the association between household food insecurity or low maternal social support and maternal common mental disorders in East Mamprusi Municipality, Ghana.

**Methods:**

This was a community-based, cross-sectional study involving 400 mothers with children 6–23 months selected using multi-stage sampling. Summary scores for household food insecurity, maternal social support, and maternal common mental disorders were measured using Food Insecurity Experience Scale (FIES), Medical Outcome Study Social Support Scale (SSS), and WHO Self-Reporting Questionnaire 20 items (SRQ-20) respectively in personal interviews. Poisson regression models were fitted to determine the association of household food insecurity or low maternal social support with maternal common mental disorders, controlling for selected socio-demographic variables.

**Results:**

The mean age of the participants was 26.7 (± 6.68) years, and the mean FIES, SSS, and SRQ-20 scores were 5.62 [95% Confidence Interval (CI): 5.29–5.96] out of 8, 43.12 (95% CI: 41.34–44.90) out of 100, and 7.91 (95% CI: 7.38–8.45) out of 19 respectively. About two-thirds of the households (71.9%), and 72.7% and 49.5% of the women had food insecurity, low social support and probable common mental disorder respectively. In the adjusted analyses, a unit increase in FIES score was associated to a 4% increment in the predicted SRQ-20 score [Incident Risk Ratio (IRR) 1.04; 95% Confidence Interval (CI): 1.02, 1.06; p = 0.001], and the predicted SRQ-20 score of the women belonging to low social support category was 38% higher compared to that of women of high social support category (IRR 1.38; 95% CI: 1.14, 1.66; p = 0.001).

**Conclusion:**

The prevalence of household food insecurity and common mental disorders among mothers are high, and both household food insecurity and low social support are significantly related to common mental disorders in women. Interventions to reduce both household food insecurity, and common mental disorders in women are warranted, and should include social support for women.

## Introduction

Common mental disorders (CMDs) include a range of anxiety, depressive, and somatoform disorders [1]. Globally, the one-year and lifetime prevalence of CMD in females were estimated at 19.8% and 32.1% respectively in a meta-analaysis [2]. According to the Institute of Health Metrics and Evaluation, about 280 million people suffer from depression at the global level [3]. The World Health Organization has indicated that over 700,000 people die from suicides annually [4], and it is projected that the leading cause of disability by 2030 would be depression [5]. CMD constitutes a significant reason for sick days and increases demand on health services making it a public health problem [6]. Depression is among the commonest mental health issues in Ghana and in the Northern region and some studies have reported the prevalence of depression in mothers to be in the range 7–33.5% [7–9].

Food has an important role in both physical and mental wellbeing and a deficiency of some micro and macro-nutrients have been implicated in the etiology of mental illness [10]. Food insecurity exists when “the availability of nutritionally adequate and safe foods or the ability to acquire acceptable foods in socially acceptable ways is limited or uncertain” [11]. About half of Ghanaians (47.7%) and 38.7% of the inhabitants of Northern Region are estimated to be food insecure in 2020 [12] and 36.0% of households are food insecure in Northern Ghana [13]. Food insecurity is considered a critical public health concern because of its devasting consequences on people from both developed and developing regions [14]. Although food insecurity affects people of all ages and sexes, its effect on women deserves more attention [15]. As a result of the mothers being the main caregivers of young children, they may be unable to participate in income generating activities and without spousal support and/or adequate savings, this can predispose them to poverty and food insecurity. Nguyen et al., [16], using a cross-sectional design in Bangladesh, Vietnam and Ethiopia revealed that, women from food insecure households or of lower socio-economic status had higher odds of CMD when compared to women from food secure households or of higher socio-economic status. Similarly, it was found that mothers from households with very low food security had higher chances of sub-optimal mental health status as well as being exposed to adverse mental health outcomes in Ecuador [17].

Children tend to be directly affected by the mental health of their caregivers since they depend on them for social and nutritional needs to develop and grow optimally. A study in Pakistan showed that within a period of 12 months, infants of depressed mothers were affected more by diarrhoea as compared to those of non-depressed mothers [18]. Another study in Ghana and Côte D’Ivoire revealed that, children of mothers with CMD symptoms were more likely to have febrile illnesses than those of mothers without CMD symptoms at 3 and 23 months postpartum [19]. A case-control study in the city of Salvador, northern eastern Brazil, found that CMD in mothers increased the risk of moderate or severe acute malnutrition in children aged 0 - 5 years [20].

The mental health status of individuals is influenced by multiple social, psychological, and biological factors [21]. Women with young children are even more at risk of poor mental health because of the physiological and social changes occurring around the perinatal period, both of which directly and indirectly influence mental well-being. CMD is associated with low educational attainment, poverty, and unemployment [6], and poor self-reported health status, smoking and chronic disease [22]. Social support is an independent determinant of mental health and wellbeing [23] but can also moderate the link between risk factors and mental illness [24]. Social support is described as the “support accessible to an individual through social ties to other individuals, groups, and the larger community” [25] or the “extent to which social relationships fill specific needs, or the degree of social integration of an individual” [26, 27].

There is limited research on the epidemiology and risk factors of mental illness in women in developing regions, and studying the risk factors of poor mental health can help identify effective interventions and reduce disease burden and impact. While household food insecurity is studied relatively frequently in Ghana, its association with poor mental health in postpartum women in Northern Ghana is less studied. Identifying household food insecurity as a risk factor may offer many opportunities for intervention in the study area and other places with similar characteristics. Therefore, this study sought to examine the association between household food insecurity or low maternal social support and CMD in mothers in East Mamprusi Municipality, Northern Ghana, while controlling for selected socio-economic and demographic characteristics. We hypothesized that there is a positive relationship between household food insecurity or low maternal social support and CMD in women in East Mamprusi Municipality.

## Materials and methods

### Study design, setting, and population

A community-based, cross-sectional study was carried out in East Mamprusi Municipality, Ghana. The East Mamprusi Municipality is in the newly created North East region and covers a land mass of 1,660sqkm. According to 2021 Population and Housing Census [28], the population of the North East region is 658,946 with 336,797 females and 322,149 males. Most of the inhabitants are young i.e., between 15 and 40 years, and are predominantly farmers, residing in rural areas. There are eleven (11) health facilities in the municipality. The study population comprised mothers with children 6–23 months in the Municipality.

### Sample size and sampling

The sample size was determined using the one-point cross-sectional sample estimation formula [29]. The sample size of 400 was determined based on the assumed proportion of mothers with CMD (assumed to be 50% to obtain the largest sample size [30]), 5% margin of error, and 95% confidence level. Study participants were selected using multi-stage sampling technique. The East Mamprusi Municipality was stratified based on the five (5) existing sub-municipalities. Simple random sampling was used to select three strata (sub-municipalities) namely Nalerigu, Sakogu and Gambaga. The sample size was proportionately allocated to the selected sub-municipalities based on population size. For each selected stratum, communities were randomly selected, a list of households within the selected communities was constructed and simple random sampling used to select households. For each selected household, an eligible woman was selected, and in cases where there was more than one eligible woman in the selected household, simple random sampling was used to select one woman. Selected households without any eligible mothers were replaced.

### Data collection

The data for the study were collected in September and October, 2020. After sampling and informed consent, personal interviews were conducted in a private area in the homes of respondents using questionnaires. Data were collected on household food security, CMD, social support, and socio-demographic and economic characteristics of respondents. The interviews were conducted in the local language spoken in the study area (Mampruli) by 6 enumerators and two supervisors. The enumerators have a minimum of bachelor’s degree in Community Nutrition and the Supervisors are lecturers in the School of Allied Health Sciences, UDS. Prior to the data collection exercise, there was a 3-day training exercise for the team of enumerators aimed at getting them to understand the contents of the questionnaires and to appreciate the survey process.

### Measurement of variables

#### Exposure variables

Household food insecurity: Household food security over the previous 12 months before the study was assessed using Food Insecurity Experience Scale (FIES) [31]. FIES is a food insecurity severity experience matrix that relies on responses of respondents to eight questions about their access to adequate food over a given period of time. Questions in this scale require respondents to answer “yes” or “no” about their access to adequate food. Each respondent’s answer was scored (“1” for “yes” and “0” for no), and the sum of the scores for the 8 questions calculated as FIES score. The FIES score theoretically ranges from 0 to 8 with a higher score depicting increased probability of food insecurity. The women were categorized into two groups i.e., food security and food insecurity (moderate or severe food insecurity) based on their responses to the 8 questions. The women who affirmed questions 4–6 i.e., skipped a meal, ate less than they thought they should have eaten, and household ran out of food were classified as moderately food insecure, and those who affirmed questions 7 and 8 i.e., felt hungry but did not eat and went without eating for a whole day were classified as severely food insecure [31]. Those who affirmed the remaining three questions or did not affirm any of the 8 questions were classified as not experiencing food insecurity. FIES was validated by FAO-VoH in Sub-Saharan Africa and has shown acceptable level of validity and reliability (Cronbach alpha 0.78) [32]. In this study, the Cronbach’s alpha of the scale is 0.98.

Social support: The Medical Outcome Study Social Support Scale (SSS) was used to measure the social support perceived to have been received by the women. SSS is a 20-item, 4-component multidimensional, self-administered instrument used to assess four aspects of social support, including tangible support, emotional-informational support, positive social interactions, and affectionate support. The subjects responded to the scale using a five-point Likert rating scale ranging from “*none of the time” (1)* to “*most of the time” (5)*. The mean scores of the overall scale was transformed into percentage using the formula: *“Percentage score = [(observed score − minimum possible score)/(maximum possible score − minimum possible score)] × 100”* [33]. The SSS score therefore ranges from 0 to 100 with a higher score corresponding to a higher level of perceived social support received by the respondents [26]. The scale, originally developed for patients with prevalent and treatable chronic conditions in the Medical Outcome Study, has a high level of reliability and validity in a validation study (Cronbach’s alpha 0.91) [26] and in the current study (Cronbach’s alpha 0.93). SSS score was used to construct a variable with two levels i.e., raw score < 50 “Low” and raw score ≥ 50 “High” categories of maternal social support based on the response distribution.

#### Outcome variable

Common Mental Disorders: CMDs or general poor mental health in mothers was measured using the Self Reporting Questionnaire 20 items (SRQ-20), which consists of 20 yes/no questions with a reference period of the previous 30 days. SRQ-20 measures several symptoms of depression, including sleep disturbance, headache, poor appetite, helplessness, depressed mood, unhappiness, and psychomotor retardation. For the first 19 questions, an affirmative answer attracts a score of “1” and the non-affirmative answer a score of “0”. The addition of the scores gives the SRQ-20 score which ranges from 0 to 19, and the higher the score, the higher the probability of CMD. A cut-off of ≥ 8 SRQ-20 score was used to identify probable cases of CMD as it was used in a number of studies on women with similar characteristics as the women in our study [34–37]. The SRQ-20 questionnaire has a high internal consistency value in the current study, Cronbach’s alpha 0.92.

#### Covariates and factors

Socio-demographic characteristics: Age in years and parity were treated as continuous variables, and most of the variables had two categories: educational status (formally educated and not formally educated); religion (Islam and Christianity); ethnicity (Mamprusi and “others”); sleeping material (mat and mattress); household main source of water (pipe and well/borehole); and household main source of lighting (electricity and others). The rest of the variables were: occupation (trader, agriculture worker, service worker, and unemployed), and residential status (rural, urban and peri-urban). Household socio-economic status was measured by asking the mothers on the ownership of 14 household items: radio, TV, satellite dish, DVD player, mobile phone, sewing machine, mattress, refrigerator, computer, electric fan, bicycle, motorcycle/tricycle, animal-drawn cart, and vehicle. Possession of an item was assigned a score of “1” otherwise a score of “0”. Principal component analysis was used to generate a household wealth score which was used to classify respondents’ households into three socio-economic groups: low, medium and high tertiles [38]. Household socioeconomic status is a composite measure of a household’s cumulative living standard.

### Data analysis

Data collected were analysed using Stata IC (Version 15) (Stata Corps, Texas). Continuous variables were presented by calculating means and standard deviations or 95% confidence intervals and categorical variables frequencies and proportions. To take into consideration the clustering nature of the data given the multi-stage nature of the survey procedure, survey weights corresponding to the reciprocal of the selection probability for each sub-municipality were used. Thus, the Stata “svy” command was used in the analysis. The association between household food insecurity and maternal CMD was evaluated using the household FIES score, and SRQ-20 score in a Poisson regression model, controlling for selected covariates and factors identified in the literature a priori as the aim was to construct an explanatory model. Similarly, the association between low maternal social support and CMD was evaluated controlling for confounders. FIES and SRQ-20 scores derived from the interviews were used as measured without any further processing (i.e., as continuous variables) in the regression models. Incident Risk Ratios (IRRs) and their 95% confidence intervals (95% CI) were computed. Statistical significance was set at p < 0.05.

### Ethics permission and consent to participate

The study was carried out in conformity to the Declaration of Helsinki. Ethics permission was obtained from the Committee on Human Research, Publications and Ethics, Kwame Nkrumah University of Science and Technology/Komfo Anokye Teaching Hospital, Kumasi, Ghana (CHRPE/AP/416/20). Written informed consent was obtained from each study participant and assent from mothers who could not read or write. At the end of the interviews, the husbands of those who could not read or write were approached to sign the informed consent forms on behalf of their spouses.

## Results

### Socio-demographic characteristics of respondents

A total of 400 mothers were involved in the study with a mean age of 26.7 (± 6.68) years and mean parity of 3.1 (± 1.89), Table 1. A greater majority of the respondents practiced Islamic religion (92.0%) or slept on mat (84.3%) but about two-thirds of them had no education (64.0%) or were Mamprusis (66.3%). Most of the mothers were involved in agricultural work (33.5%) or were unemployed (32.0%) and majority (57%) resided in peri-urban areas. With respect to the household characteristics, most of the respondents belonged to lower socioeconomic households (33.8%), or households that depended on well/borehole for water (57.3%) and electricity for lighting (80.3%).

**Table 1:** Socio-demographic and household characteristics of respondents in East Mamprusi Municipal, Ghana

Variables	Frequency (Percent)/ Mean (±Standard deviation)
Age (years)	26.7 (± 6.68)
Number of births	3.1 (± 1.89)
Educational status	
Not educated	256 (64.0)
Educated	144 (36.0)
Ethnic group	
Mamprusi	265 (66.3)
Others	135 (33.8)
Religious affiliation	
Christianity	32 (8.0)
Islam	368 (92.0)
Occupation	
Trader	76 (19.0)
Agriculture worker	134 (33.5)
Service worker	62 (15.5)
Unemployed	128 (32.0)
Sleeping material	
Mat	337 (84.3)
Mattress	63 (15.8)
Household socio-economic status (tertile)	
Low	135 (33.8)
Medium	139 (34.8)
High	126 (31.5)
Household main source of water	
Pipe water	170 (42.5)
Well/borehole	230 (57.5)
Household main source of lighting	
Electricity	323 (80.3)
Others	77 (19.3)
Residential status	
Rural	87 (21.8)
Urban	84 (21.0)
Peri-urban	229 (57.3)

### Association between household food insecurity or low maternal social support and maternal common mental disorders

The mean FIES, SSS, and SRQ-20 scores were 5.62 (95% Confidence Interval (CI): 5.29–5.96) out of 8, 7.91 (95% CI: 7.38–8.45) out of 19, and 43.12 (95% CI: 41.34–44.90) out of 100 respectively, Table 2. About two-thirds of households (71.9%), and 72.7% and 49.5% of the mothers had food insecurity, low social support and probable CMD respectively. Both unadjusted and adjusted Poisson regression models were produced to study the association of household food insecurity or low maternal social support with CMD in mothers while controlling for selected socio-demographic characteristics. Models 1–8 in Table 3 show the crude effect estimates of the association between the two exposure variables (FIES score and maternal social support) and SRQ-20 score and the association between the six (6) potential confounders and SRQ-20 score. In the unadjusted analysis, a unit increase in FIES score was associated to a 6% increment in SRQ-20 score [Incident Risk Ratio (IRR) 1.06; 95% Confidence Interval (CI): 1.04, 1.09; p < 0.001] but this reduced to 4% after adjustment for confounders (IRR 1.04; 95% CI: 1.02, 1.06; p = 0.001), Table 3. For the association between maternal social support and CMD, it was found that receiving low social support was associated with 60% and 38% increments in SRQ-20 score compared to high social support in the unadjusted model (IRR 1.60; 95% CI: 1.31, 1.96; p < 0.001), and adjusted model (IRR 1.38; 95% CI: 1.14, 1.66; p = 0.001) respectively.

**Table 2:** Summary FIES, SSS, and SRQ-20 scores, and proportions of households with food insecurity, and women with probable CMD in East Mamprusi Municipal, Ghana

Variable	Mean/Proportion	Standard error of mean	95% Confidence Interval of mean/proportion
Food Insecurity Experience Scale score	5.62	0.17	5.29–5.96
Social Support Scale score	43.11	0.91	41.34–44.90
Self-Reporting Questionnaire-20 items score	7.91	0.27	7.38–8.45
Women with low social support	72.7		68.1–77.0
Households with food insecurity	71.9		67.6–76.3
Women with probable CMD	49.5		44.9–54.0

**Table 3:** Association between Food Insecurity Experience Scale score or Maternal Social Support class and Self Reporting Questionnaire-20 score of mothers in East Mamprusi Municipal, Ghana

	Unadjusted Models with Covariates and Factors (Models 1–8)		Adjusted Model with Food Insecurity Experience Scale score as exposure (Model 9)		Adjusted Model with Maternal Social Support status as exposure (Model 10)	
Independent Variable	Incident Rate Ratio and 95% Confidence Interval	P-value	Incident Rate Ratio and 95% Confidence Interval	P-value	Incident Rate Ratio and 95% Confidence Interval	P-value
Educational status (Ref: Educated)						
Not educated	1.34 (1.14, 1.58)	0.001	1.19 (1.02, 1.39)	0.031	1.21 (1.03, 1.42)	0.021
Parity	1.01 (0.98, 1.05)	0.492	0.99 (0.96, 1.03)	0.724	1.00 (0.96, 1.03)	0.849
Occupation (Ref: Trader)						
Agric worker	1.49 (1.17, 1.89)	0.001	1.20 (0.97, 1.49)	0.088	1.23 (0.99, 1.52)	0.063
Service worker	1.16 (0.87, 1.54)	0.314	1.10 (0.87, 1.41)	0.421	1.10 (0.87, 1.41)	0.424
Unemployed	1.36 (1.06, 1.75)	0.016	1.18 (0.95, 1.45)	0.126	1.20 (0.97, 1.48)	0.101
Received support with childcare (Ref: Yes)						
No	1.48 (1.27, 1.73)	< 0.001	1.47 (1.28, 1.68)	< 0.001	1.46 (1.28, 1.68)	< 0.001
Household socio-economic status (Ref: High tertile)						
Low tertile	1.47 (1.21, 1.79)	< 0.001	0.97 (0.81, 1.17)	0.758	1.02 (0.84, 1.24)	0.847
Medium tertile	1.40 (1.15, 1.72)	0.001	1.01 (0.84, 1.22)	0.905	1.05 (0.87, 1.28)	0.588
Residential area (Ref: Urban)						
Rural	2.15 (1.58, 2.94)	< 0.001	1.79 (1.32, 2.43)	< 0.001	1.86 (1.37, 2.54)	< 0.001
Peri-urban	2.22 (1.68, 2.95)	< 0.001	1.63 (1.24, 2.16)	0.001	1.73 (1.30, 2.29)	< 0.001
Maternal social support (Ref: High)						
Low	1.60 (1.31, 1.96)	< 0.001	1.36 (1.13, 1.64)	0.001	1.38 (1.14, 1.66)	0.001
Food Insecurity Experience Scale score	1.06 (1.04, 1.09)	< 0.001	1.04 (1.02, 1.06)	0.001		

Model 9 controlled for educational status, parity, occupation, support with childcare, household socio-economic status, residential area, and maternal social support.

Model 10 controlled for educational status, parity, occupation, support with childcare, household socio-economic status, and residential area.

## Discussion

This study sought to examine the association of household food insecurity or low maternal social support with CMD in mothers in East Mamprusi Municipality, Ghana. A higher proportion of households and mothers have food insecurity and probable CMD respectively. Maternal CMD risk is related to both household food insecurity and low maternal social support, even after adjusting for potential confounders.

### Prevalence of household food insecurity and CMD

Almost two-thirds of the households have food insecurity, making household food insecurity widespread in the district, but the Feed the Future baseline survey found a lower prevalence of household food insecurity (36%) in northern Ghana [13]. The disparity in these prevalence figures could be due to differences in socio-economic status of the populations studied. The prevalence of household food insecurity has been estimated to range from 7.5 to 73.0% in developing countries [39]. Poverty [40, 41] and low socio-economic status [42–45] have been linked to household food insecurity. According to the Ghana Poverty Map [46], nearly half of the people in the East Mamprusi area are poor. The recent covid-19 pandemic might also have increased the scarcity of food and subsequently driven up demand for food and food prices. Several studies have reported that the pandemic had a negative effect on food security and nutrition in the West African sub-region e.g., [47, 48]. In Ghana, it was found that the pandemic negatively affected wage and total household incomes and secondarily negatively affected food security [49]. The high poverty level and low socio-economic status of households, and high food prices meant the households possibly would not have had the purchasing power to ensure that food was always available and accessible to household members, thereby making food insecurity prevalent in the Municipality.

The prevalence of CMD in mothers measured in the current study (49.5%) is closer to prevalence of 41.0% in Nigeria [34], and 46.2% in Bangladesh [50], underscoring the widespread surge in CMDs in women. Our prevalence is however higher than the 20.5% reported for a cohort of women in Brazil [36]. The high prevalence of CMD measured may also be a result of the high poverty level and low socio-economic status of households in the municipality. A study by Knifton & Inglis [51] highlights the direct relationship between poverty and mental illness and eventual health inequalities, and low socio-economic status has been found to associate with CMD [36, 52–54]. The covid-19 pandemic might have also contributed to the prevalence of CMD in the mothers, probably due to lack of social safety nets for citizens during the lockdown, presence of covid-19-related psychological distress, disruption of social patterns, and negative impact on incomes.

### Association between household food insecurity and CMD

Household food insecurity was shown to be significantly associated with CMD in the current study even when social support and other variables were adjusted for. This observation is in line with the findings of other studies in which maternal mental health status was significantly associated with food security [37, 52, 55, 56]. Concerns over provision of food or household food insecurity or inability to afford basic needs including food may increase cognitive load acting as a stressor and increasing the risk of CMD in mothers [57, 58]. The association between household food insecurity and poor mental health may be bidirectional [59]. It is possible that having CMD could lead to an increase in the risk of household food insecurity. Mothers with mental disorders may have lowered chances of landing permanent jobs or may expend more resources to address health problems, resulting in household food insecurity [17, 52, 59, 60].

### Association between low maternal social support and CMD

We found a higher mean SRQ-20 score for women with low social support compared to those with high social support even after adjusting for important socio-demographic characteristics, suggesting that social support is protective of poor mental health. Positive associations between social support and psychological well-being [61, 62] as well as inverse associations between social support and depression [62, 63] have been reported. While the exact mechanism by which social support influences mental health positively is unknown, two mechanisms have been proposed: the stress-buffering model and the main effects model [64]. The stress-buffering model suggests that perceived availability of social support eliminates or weakens the relationship between a stressor and poor mental health and makes available resources for coping with stress whereas the main effects model postulates that social support and social network provide a sense of belonging and stability resulting in improved self-esteem and lower risk of mental illness.

### Strengths and limitations of the study

The main strength of this analysis is the use of three widely validated international scales for data collection. The analysis also treated the outcome variable as a continuous variable and made use of Poisson regression modelling which is not so common in the literature. With respect to limitations, the cross-sectional nature of the study could not allow for causality assessment, and the SSS was developed for patients with chronic diseases and not validated for women in low and middle income countries. We also measured food insecurity at the household level instead of at the individual level since what is available for the household may not be equally shared among the household members. Nonetheless, this study provides important data on prevalence and determinants of maternal CMD, which will inform design of interventions to reduce the burden and severity of this condition in the study area and other areas having similar characteristics as the study area.

## Conclusion

Higher proportion of both households and mothers have food insecurity and probable CMD respectively, and both household food insecurity and low maternal social support are associated with the CMD status of the women. These findings add to the body of evidence linking household food insecurity and low social support to maternal CMD, and provide information for healthcare policy makers and other stakeholders to develop appropriate strategies, interventions and programmes, to support sound mental health and improved well-being of mothers and children.

## Data Availability

The datasets used and/or analysed during the current study are available from the corresponding author on reasonable request.

## List of abbreviations

CMDs: Common Mental Disorders
FIES: Food Insecurity Experience Scale
IRR: Incident Risk Ratio
SSS: Social Support Scale
SRQ-20: Self-Reporting Questionnaire 20 items
WHO: World Health Organisation
